# Assessment of treatment-induced female sexual morbidity in oncology: is this a part of routine medical follow-up after radical pelvic radiotherapy?

**DOI:** 10.1038/bjc.2011.339

**Published:** 2011-09-06

**Authors:** I D White, H Allan, S Faithfull

**Affiliations:** 1 Supportive Cancer Care Research Group, Florence Nightingale School of Nursing and Midwifery, James Clerk Maxwell Building, King's College, 57 Waterloo Road, London SE1 8WA, UK; 2 Division of Health and Social Care, Faculty of Health and Medical Sciences, University of Surrey, Stag Hill, Guildford GU2 7TE, UK

**Keywords:** pelvic radiotherapy toxicity, sexual morbidity, assessment

## Abstract

**Background:**

Oncology follow-up has traditionally prioritised disease surveillance and the assessment and management of symptoms associated with cancer and its treatment. Over the past decade, the focus on late effects of treatment has increased, particularly those that have an adverse effect on long-term function and quality of life. The aim of this research was to explore factors that influence the identification of treatment-induced female sexual difficulties in routine oncology follow-up after radical pelvic radiotherapy.

**Methods:**

A structured observation schedule was used to systematically record topics discussed in 69 radiotherapy follow-up consultations observed over a 5-month period.

**Results:**

Analysis suggests that physical toxicity assessment focused on bowel (81%) and bladder (70%) symptoms. Vaginal toxicity was discussed less frequently (42%) and sexual issues were explored in only 25% of consultations. Formal recording of radiation toxicity through assessment questionnaires was limited to patients participating in clinical trials. Surveillance activity and the management of active physical symptoms predominated and psychosocial issues were addressed in only 42% of consultations.

**Interpretation:**

Female sexual morbidity after pelvic radiotherapy remains a neglected aspect of routine follow-up and cancer survivorship. Developments in both individual practice and service provision are necessary if the identification and management of treatment-induced female sexual difficulties is to be improved.

Assessment of treatment late effects has received considerable attention over the past decade, thus acknowledging the increased numbers of people living with their adverse impact (Creutzberg *et al*, 2000; Rowland *et al*, 2006; Barker *et al*, 2009; Ganz, 2009). There are approximately 1.8 million people in the United Kingdom recognised as cancer survivors (Department of Health, 2010, 2011) and an estimated 1 in 10 women living with a cancer diagnosis have been treated for a gynaecological malignancy (Forman *et al*, 2003).

Evaluating morbidity associated with cancer treatment has relied predominantly on clinician reports and observer data, and late effects in particular have tended to be under-recognised and under-reported (Davidson and Faithfull, 2006; Andreyev *et al*, 2010). Furthermore, the time course and slow trajectory of onset for many radiotherapy late effects (months to years) may further impede their recognition and treatment by clinicians (Rowland *et al*, 2006; Barker *et al*, 2009; Ganz, 2009). Recent advances in morbidity assessment within oncology include the use of patient reported outcome measures (PROMs) to improve patient experience and continuity of care (Velikova *et al*, 2010). From a clinician's perspective, recognising specific late effects may be influenced by a range of factors, including experience, training, time constraints and whether or not interventions are readily available for the problem(s) identified (Murphy, 2009). Accurate assessment and recognition are therefore the first steps in appropriately managing adverse effects.

There is a now a growing realisation from policy makers, clinicians and patient groups that the assessment and management of treatment consequences must be more effectively addressed through innovation in both research and service delivery (Macmillan, 2008; Maher and Denton, 2008; Andreyev *et al*, 2010; Department of Health, 2010, 2011).

Each year in the United Kingdom approximately 17 000 people receive pelvic radiotherapy for the treatment of gynaecological, ano-rectal, bladder or prostate cancer (West and Davidson, 2009). Treatment late effects related to the dose distribution of pelvic radiotherapy can have a negative impact on quality of life for at least 2 years after acute radiation effects have diminished (Barker *et al*, 2009).

Pelvic radiotherapy is associated with bowel and bladder toxicity, loss of fertility, vaginal and sexual changes. Yet, while radiation-induced bowel and bladder toxicity are commonly reported, detail of radiotherapy-induced female sexual morbidity is often more limited (Maher and Denton, 2008; Barker *et al*, 2009).

Estimates of the prevalence of female sexual difficulties after pelvic radiotherapy vary markedly depending on problem definition, scope and validity of research instruments used. Studies of sexual difficulties associated with radiotherapy for cervical cancer indicate prevalence rates of between 30 and 80% (Flay and Mathews, 1995; Bergmark *et al*, 1999; Davidson *et al*, 2003; Jensen *et al*, 2003; Vistad *et al*, 2006). Furthermore, despite a plethora of patient-rated QOL studies in long-term survivors of cervical cancer (Vistad *et al*, 2006), there remains a paucity of good quality research exploring the sexual recovery of women after endometrial, bladder, rectal or anal cancer.

Sexual well-being is acknowledged as a core aspect of quality of life for people affected by cancer, particularly those receiving treatment for pelvic malignancies (Flay and Mathews, 1995; Wenzel *et al*, 2005; Vistad *et al*, 2006; Foster *et al*, 2009). Physical effects include vaginal dryness, fibrosis, stenosis or shortening, vaginal bleeding and discharge, menopausal symptoms, skin reactions, urinary difficulties, disruption to bowel function and infertility (Jeffries *et al*, 2006; Maher and Denton, 2008). However, common psychological responses include anxiety, depression, fear of sexual pain and altered femininity.

Findings from recent radiotherapy morbidity studies appear to indicate that women receiving primary or adjuvant pelvic radiotherapy experience greater and more prolonged disruption to their sexual well-being (Davidson *et al*, 2003; Jensen *et al*, 2003) than women after surgery alone (Leake *et al*, 2001; Juraskova *et al*, 2003).

Yet, despite increasing evidence of the organic basis for female sexual morbidity after pelvic radiotherapy, the assessment and management of treatment-induced sexual difficulties remains frequently overlooked in routine cancer follow-up. Health professionals experience difficulty discussing sexual aspects of treatment (Jensen *et al*, 2003; Stead *et al*, 2003), resulting in ongoing distress for women even when physical problem(s) have diminished.

The first step towards being able to treat and manage the consequences of cancer treatment is clinical assessment (Richardson *et al*, 2006); without knowing the prevalence of symptoms and problems, it remains difficult to identify and meet patient needs effectively. Hence, the principal aim of this study was to explore the factors that influence the assessment of female sexual difficulties as a treatment consequence within routine oncology follow-up.

## Materials and methods

We report results from observation of follow-up clinics with health professionals, patients and partners. These data originate from a larger mixed method study that included in-depth interviews exploring patient, clinician and organisational factors that influence the discussion of sexual morbidity in oncology consultations. Qualitative analysis of interview data is reported elsewhere. Structured observation of radiotherapy outpatient clinics allowed the nature and content of assessment undertaken by clinicians to be described and the reality of medical follow-up practice to be analysed.

Observation data were collected from three gynaecological and two colorectal radiotherapy clinics within two South of England cancer centres. Consultations with women who had a diagnosis of cervical, endometrial anal or rectal cancer treated by radical pelvic radiotherapy from 6 weeks to 2 years previously, attending routine medical follow-up, met study inclusion criteria and were invited to take part in the study. A specific clinician was shadowed for the clinic duration to minimise the researcher's influence on routine clinic processes.

Approval for the study was obtained from the Local Research Ethics and Research and Development committees of both NHS Trusts. Patients and health professionals in outpatient consultations gave verbal consent to participate in the observation element of the study. Study information sheets were given to patients in advance of their appointment time to ensure they felt able to give informed consent for the researcher to be present during their consultation. Verbal consent to participate in the study was sought by the clinician conducting the consultation before the researcher entered the consulting room. No patients refused to take part in the study, but the researcher was asked to remain outside the consultation room on four occasions where their presence was considered inappropriate, for example, breaking bad news.

As it was important to observe normal practice during consultations, the specific topic of the study remained covert in order not to influence patient-led discussions or agenda setting. Medical staff conducting follow-up were, however, aware of the study topic in advance of granting permission for the researcher to be present.

An observation schedule enabled rapid recording of patient demographics, topics discussed and identification of the initiator of each topic. Topics included: bowel, bladder and vaginal toxicity, skin reactions, pain, other symptoms, medication, test results, future treatment and follow-up plans, psychosocial issues and sexual issues.

Data analysis was undertaken using SPSS (v.14, IBM, New York, NY, USA) to explore the frequency and range of topics discussed and to identify any relationship between topic prevalence and participant demographics. Pearson's *χ*^2^-test was used to explore the relationship between sets of categorical data in a series of contingency tables. The *χ*^2^-statistic, Fisher's exact test, degrees of freedom (d.f.) and significance values were reported for each variable comparison conducted.

## Results

A total of 141 individual consultations from 31 separate gynaecological and colorectal radiotherapy clinic sessions were observed over a 5-month period. In all, 72 (51.06%) consultations were subsequently excluded as they did not meet the study entry criteria (male patients, excluded primary diagnoses/treatments).

Data analysis was based on 69 (48.94%) observed consultations with women who met the study entry criteria. Medical staff conducting follow-up included five consultants, five specialist registrars and one clinical research fellow. Of the 69 consultations observed, 43 (62.3%) were conducted by male clinicians and 26 (37.7%) with female clinicians.

A summary of the demographic details of patients participating in observed consultations is presented in Table 1. The majority of women (*n*=50, 72.5%) had a diagnosis of cervical or endometrial cancer and were aged over 60 years (*n*=37, 53.6%). The sample included women with both early (*n*=29, 43.3% clinical stage I/II) and late stage (*n*=38, 56.7% clinical stage III/IV) disease who had received radical radiotherapy in the management of their illness. Consultations at different time points in the women's follow-up period were also sampled.

The sample was broadly representative of the age range of women affected by these cancer types in the UK population. However, in this study women with cervical (*n*=20, 29%) and anal cancer (*n*=5, 7.2%) were over-represented (Cancer Research UK, 2011).

None of the study participants were receiving experimental treatments, although 6 out of 19 women with anal or rectal cancer were enrolled in clinical trials requiring toxicity monitoring that included vaginal and sexual morbidity. Both study sites receive secondary and tertiary referrals for radical pelvic radiotherapy.

The majority of women had a current partner (*n*=48, 69.6%), although details of relationship status were missing for seven (10.1%) women. Relationship details were taken from the women's medical records and all women were noted as being in a heterosexual relationship.

**Figure 1:**
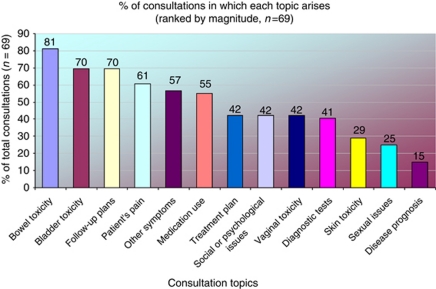
Range of topics discussed during women's consultations with medical staff.

The most frequently discussed consultation topics related to the impact of pelvic radiotherapy on both bowel and bladder function in 81% (*n*=56) and 70% (*n*=48) of consultations, respectively (Figure 1). However, no formal method of toxicity recording was used by practitioners unless the woman was enrolled in a clinical trial where toxicity data sheets were used (six women with anal or rectal cancer/8.7% of total observed consultations).

There were 60 *other* topics discussed, the majority of which were physical side effects of treatment ranging from anorexia, nausea, dietary intake or weight gain, to concerns about fatigue, general weakness, lymphoedema and continence management in 39 out of 69 (57%) of the consultations.

Psychological or social aspects of the women's illness and treatment were discussed in 42% (*n*=29) of the consultations and varied from in-depth discussion of the impact of disease recurrence on family or sources of social and emotional support to brief enquiries as to whether or not the woman had returned to work following treatment completion.

Health-care professionals were dominant in leading discussions for all of the 13 topics summarised in Figure 1, with women initiating topic discussions with their treatment team in fewer than 14.5% of consultations.

### Discussion of vaginal toxicity

Radiotherapy-induced vaginal toxicity was discussed in 29 (42%) of the consultations observed. Vaginal symptoms were either elicited by verbal questioning or through vaginal examination. Vaginal examination was a routine aspect of medical review in the gynaecological clinics, but performed only when clinically indicated by reports of vaginal symptoms in the colorectal clinics.

Vaginal bleeding was the symptom most commonly raised in 17 consultations by both clinicians (*n*=13, 18.8%) and women (*n*=4, 5.8%). Despite the prominence of vaginal stenosis, vaginal shortening and vaginal dryness in the biomedical literature as common toxicities after pelvic radiotherapy, these vaginal symptoms were only discussed in 11 (15.9%), nine (13%) and six (7.2%) observed consultations, respectively. Manifestations of radiation-induced vaginal changes such as telangectasia or ulceration were normally discussed with women only when they resulted in vaginal bleeding; otherwise, they were simply noted in patient's records.

There was no statistically significant relationship between the discussion of vaginal symptoms and the time elapsed since women had completed their radiotherapy treatment. Vaginal symptoms were prevalent in 10 out of 31 (32.2%) women who were less than 6 months post-treatment, in 5 out of 9 (55.5%) women who had completed treatment 6–11 months previously and in 14 out of 29 (48.2%) women at 12 months or more post-treatment.

The provision of vaginal dilators as a prophylactic intervention to reduce the likelihood of developing vaginal stenosis and shortening associated with pelvic radiotherapy was standard practice at both research sites (Miles and Johnson, 2010). Despite routine dilator provision, discussion of vaginal dilator use was absent from the majority (*n*=53, 76.8%) of medical consultations observed. There was, however, a statistically significant association (*χ*^2^=22.870, d.f.=1, *P*=0.000) between the elicitation of vaginal toxicity in consultations and a subsequent discussion of the use of dilators. In only one consultation out of 15 was there a discussion of dilator provision where vaginal toxicity had not been identified during the consultation.

Discussion of treatment-induced menopause and its management was a topic discussed in nine (13%) consultations, raised predominantly by clinicians (*n*=7, 10.1%). Although this frequency was low, it was noted that in 37 out of 69 (53.6%) consultations the woman was naturally post-menopausal at the time of her treatment, resulting in omission of this topic within 23 (33.3%) consultations where the woman had experienced a treatment-induced menopause.

### Discussion of treatment-induced sexual issues

**Figure 2:**
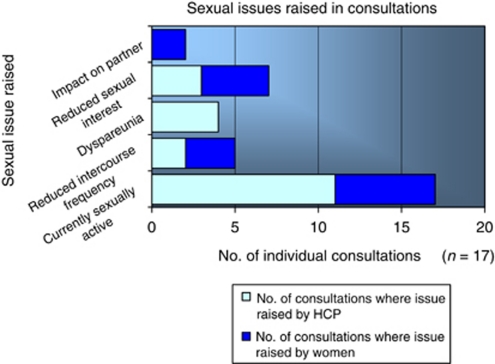
Sexual issues discussed in follow-up consultations with medical staff.

As can be seen in Figure 2, sexual issues were discussed in a total of 17 (24.6%) consultations, with health-care professionals raising this topic on 11 (15.9%) occasions and women on a further six (8.7%) occasions.

The duration of consultations ranged from 15 to over 30 min and was not only influenced by the number of topics discussed, but also the nature of consultation content regarding its complexity, significance and emotional impact for the woman in question. Analysis of the number of topics per consultation was undertaken to ascertain whether or not sexual issues were less likely to be discussed where there were a large number of current problems/issues to address. Data were grouped into consultations with a high number of topics (6–10 topics per consultation) and those with a low number of topics (2–5 topics) as there were no consultations with a topic count of less than 2 or in excess of 10. There was no statistically significant difference observed between the groups, with sexual issues equally likely to occur in consultations where the topic count was high as it was in those where fewer topics were discussed (^*^χ^2^=0.592, d.f.=1, *P*=0.442, Fisher's exact test (one-sided), *P*=0.313).

The discussion of sexual issues did not appear to be influenced by whether or not the woman had a current partner, with 12 out of 17 (70.5%) women in a current relationship compared to 5 out of 17 (29.4%) of women without a current partner. The proportion of women with or without a current partner in this subgroup of consultations where sexual issues were discussed was comparable to those in the overall sample of observed consultations (*n*=69) with 48 out of 62 (77.4%) women in a current relationship, 14 out of 62 (22.6%) not in a current relationship and seven out of 69 (10.1%) missing data where the relationship status of the woman was unknown.

In all, 30 women (43.5%) were accompanied at their consultation by a partner, adult child or a friend, but there was no statistically significant relationship demonstrated regarding whether or not the woman was accompanied at her consultation and the topics discussed in those consultations.

Another factor that did not appear to affect the discussion of sexual issues was the gender of the clinician. In the 17 consultations where sexual issues were raised by either the clinician or patient, the clinician was female in 6 out of 26 (23%) consultations and male in 11 out of 43 (25.5%) consultations. There was also no relationship between the clinician's experience in oncology/grade and whether or not sexual issues were discussed with patients.

Enquiry about a woman's sexual recovery was normally introduced by clinicians asking a direct question such as: ‘Are you sexually active at present?’ The range of sexual topics discussed during consultations between women and their treatment team was limited, with low sexual desire (*n*=7, 10.1%) and reduced frequency of intercourse (*n*=5, 7.2%) featuring more often than the discussion of dyspareunia (*n*=4, 5.8%).

Changes in orgasm were not discussed, nor was there any enquiry as to whether or not treatment had influenced women's level of sexual satisfaction. Concerns relating to partner adjustment to changes in the couple's sexual relationship associated with the woman's cancer treatment were raised by two (2.9%) of the women. Discussion of sexual issues normally focused on treatment induced changes to women's sexual function without reference to her sexual relationship other than to ascertain the presence or absence of a current partner. Onward referrals for management of treatment-induced menopause, vaginal toxicity or sexual issues arising from discussion of sexual difficulties occurred on only eight occasions. Women were referred to the radiotherapy nursing service on seven (10.1%) occasions for the discussion of dilator use, to a clinical nurse specialist in gynae-oncology on one (1.4%) and to a woman's GP on one (1.4%) occasion to discuss menopause and HRT.

The age of women appeared to influence clinician's behaviour independently of other factors. The majority of women (*n*=21 out of 69, 30.4%) fell into the 61–70 years age group, with a range from 31 to over 80 years of age. For comparative analysis, age categories were collapsed to women >60 years (37, 53.6%) *vs* those 60 years or younger (32, 46.4%). Sexual issues were more likely to be discussed with women who were younger than 60 years of age (13 out of 32 consultations) compared to those older than 60 years of age (4 out of 37 consultations), regardless of their diagnosis or the time-elapsed post-treatment (*χ*^2^=8.215, d.f.=1, *P*=0.004; Fisher's exact test (one-sided), *P*=0.005).

Another factor that appeared influential in determining whether or not sexual issues were discussed in the clinical setting was the clinical stage of the patient's illness. Women with stage I/II disease (11 out of 29; 37.9%) were more likely to have discussion of sexual concerns with their doctor than women with clinical stage III/IV disease (6 out of 38; 15.7%). The difference in these two groups was statistically significant (^*^χ^2^=4.258, d.f.=1, *P*=0.039, Fisher's exact test (one-sided), *P*=0.038) at the 5% level.

## Discussion

The aim of this study was to elicit the factors that influence the frequency and extent of enquiry about the sexual consequences of pelvic radiotherapy in routine follow-up consultations with women post-pelvic radiotherapy. Consistent with findings from published studies, standardised assessment instruments for acute radiation toxicity and late effects recording were not in routine use at either research site (Davidson *et al*, 2002, 2003; Dische and Saunders, 2003). As can be seen from Figure 1, bowel and bladder toxicity were assessed during the majority of consultations (81% and 70% of consultations, respectively) compared to only 42% of consultations where vaginal toxicity was discussed. These findings are similar to audit data from Denton *et al* (2000), whereby assessment focused on urological and bowel toxicity as late effects after radical radiotherapy for carcinoma of the cervix, with the relative neglect of vaginal toxicity and sexual morbidity.

In this study, the dominant topics discussed were treatment- or illness-related physical effects, future treatment and follow-up plans. Psychological and social topics were discussed in a minority of consultations (*n*=29, 42%), consistent with findings from a recent survey by Macmillan Cancer Support (2006), where 58% of respondents felt that cancer services addressed their emotional needs less effectively than their physical needs, despite 45% of respondents stating that the emotional effects of cancer were the most difficult to cope with.

Discussion of the sexual consequences of pelvic radiotherapy in only 17 (24.6%) observed consultations must be considered low given the 50–80% estimated prevalence of sexual difficulties following radiotherapy for gynaecological malignancy cited in published studies (Crowther *et al*, 1994; Flay and Mathews, 1995; Jensen *et al*, 2003). The rate of enquiry about the sexual consequences of pelvic radiotherapy among the sample of women in this study can also be considered low when compared to data emanating from a similar study conducted in two prostate cancer clinics (Forbat *et al*, 2011). Consultations with 60 men attending surgical or radiotherapy follow-up clinics after treatment for prostate cancer were observed over a period of 18 weeks. The mean age of men attending these clinics was 70 years, with a slightly lower median age of 65 years among men attending the surgical clinic. As in this study, sexual function was predominantly raised by clinicians (*n*=22, 39%) with discussion of sexual issues occurring in a total of 32 (53.3%) observed consultations compared to no discussion in 28 (47%) of the 60 clinic consultations observed.

The lack of clinical time to address psychological, social and sexual aspects of patient's illness experience has been previously identified as a common reason for the persistent low profile of sexual rehabilitation within health-care practice (Guthrie, 1999; Gott *et al*, 2004). During the study period, clinic volume was high and it was not uncommon for clinics to over-run. It could be argued that medical follow-up clinics are not a suitable environment for the detailed discussion or assessment of female sexual difficulties after cancer treatment due to time constraints, lack of privacy and a necessity for topic prioritisation.

As can be seen from the data, exploration of sexual issues observed within oncology follow-up clinics revealed a restricted view of female sexuality, with emphasis predominantly on the woman's ability to achieve vaginal intercourse (Hyde, 2007). Recent studies of sexual morbidity in oncology offer a more comprehensive exploration of the impact of treatment on all phases of the human sexual response cycle (Masters and Johnson, 1966). These include changes in sexual interest, physiological elements of sexual arousal (vaginal lubrication, absence of dyspareunia) and orgasmic capacity (Andersen *et al*, 1997; Kylstra *et al*, 1999). The majority of these studies also explored whether women's sexual satisfaction post-treatment compared favourably with their pre-diagnosis sexual well-being (Juraskova *et al*, 2003; Marijnen *et al*, 2005; Pieterse *et al*, 2006).

In this study, clinician discussions focused solely on the woman, with no enquiry regarding the impact of sexual changes on the partner or couple. Only two women in observed consultations raised concerns about reduced sexual interest and intercourse frequency on their partner's sexual enjoyment. This lack of focus on the couple relationship in consultations mirrors findings from biomedical literature, with only a minority of gynaecological studies (Van De Wiel *et al*, 1990; DeGroot *et al*, 2005) specifically exploring the sexual impact of cancer treatment on the partner or couple.

Health professionals report feeling uncomfortable, and perceive patients to be uncomfortable, in opposite gender consultations (Burd *et al*, 2006). The number of consultations conducted by female health professionals where sexuality was discussed was low in this study (*n*=6, 23%), and the observation method does not permit researcher exploration of clinician or patient comfort regarding the topics discussed in clinic. In this study, the gender of the clinician did not appear to influence the discussion of sexual concerns, with a discussion rate of 6 out of 26 (23%) consultations where the clinician was female and 11 out of 43 (25.5%) consultations where the clinician was male. This was a factor specifically explored within this study's interview data and is reported elsewhere.

Burd *et al* (2006) also found that doctors experienced greater ‘discomfort’ in discussing sexual issues with patients who were aged over 60 years, currently without a partner and with lower education levels. The influence of patient age on the prevalence of sexual discussions was also an important finding in this study where sexual issues were more likely to be discussed with women who were younger than 60 years of age (13 out of 32 consultations) compared to those older than 60 years of age (4 out of 37 consultations), regardless of their diagnosis or the time-elapsed post-treatment.

A study by Gott *et al* (2004) found that doctors frequently used discussion of contraception or reproductive health as a vehicle for broaching the more sensitive topic of sexual well-being among their patients. In discussions with post-menopausal women, where this strategy was not possible, sexual issues were less likely to be addressed. Gott *et al*'s (2004) findings may explain the relationship between discussion of vaginal toxicity, vaginal dilator use and the subsequent discussion of sexual issues in consultations within this study. Doctors appear to find it easier to discuss vaginal symptoms and dilator use as a means to subsequent enquiry about sexual recovery post-treatment as opposed to raising such sensitive topics directly.

The apparent reticence by many health professionals to discuss the sexual consequences of cancer treatment with women may also be partly explained by the lack of biomedical interventions developed to treat female sexual difficulties compared to those available for the management of erectile dysfunction (Miles *et al*, 2007). However, patients often gain considerable benefit simply from having their treatment-induced sexual difficulty acknowledged and legitimised by clinicians as worthy of attention even if there is no immediate treatment to offer.

### Study limitations

The observation method adopted in this study was that of a structured observation schedule compiled using an expert panel and literature review. Hence, only limited qualitative coding, identification of discussion initiator and quantitative topic counts were possible. Furthermore, as the consultations were not audio-taped, it was not possible to independently verify accuracy of the observation schedules completed by a single researcher. However, use of a structured observation schedule did promote ease and speed of topic recording during brief, busy medical consultations.

In interpreting the actual prevalence of discussions of sexual issues within 17 of the 69 observed consultations, it is important to note that all clinicians were made aware of the study focus in advance of their study participation and hence this may have influenced the rate of enquiry observed.

## Conclusion

Analysis of observation data from oncology clinics provides insight into the challenge of addressing the sexual aspects of women's recovery after pelvic cancer in the busy clinical environment of routine medical follow-up. These findings suggest that in the two cancer centres where this research took place, the clinical assessment of female sexual difficulties was not a core element of routine medical follow-up after radical radiotherapy for women with pelvic malignancies. Women experienced a lower level of enquiry about their psychosocial and sexual recovery during follow-up than attention paid to other aspects of their physical recovery and disease surveillance.

Clearly when considering the clinical implications of these findings, the need for appropriate disease surveillance and management of women's fear of disease recurrence remains of paramount importance. However, traditional models of oncology follow-up may also mean that the provision of psychosocial and sexual aspects of recovery and rehabilitation frequently remain neglected. Specialist nurses and therapy radiographers are increasingly engaged in the development and delivery of end of treatment, survivorship and late effects services. They may be able to offer greater clinician continuity for discussion of sensitive topics such as menopause, vaginal health strategies and sexual recovery and be able to support such discussions to take place in less time-limited environments using both face to face and telephone delivery formats.

Improving care for people living with and beyond cancer has received increased policy attention in the United Kingdom as a consequence of the launch of the National Cancer Survivorship Initiative (NCSI) by the Department of Health and Macmillan Cancer Support (2010). Table 2 offers some practical recommendations based on the principles enshrined within the NCSI Vision document (2010) that treatment teams and cancer centres may wish to implement to address this practice and organisational deficit.

The introduction of structured morbidity assessment and PROMS to oncology clinics has been found to improve patient satisfaction with continuity of care and clinician communication in oncology consultations (Velikova *et al*, 2010). It may be that offering a structured patient self-report questionnaire such as Lent Soma, in routine oncology practice, would assist both women and clinicians to open a more comprehensive dialogue about important pelvic late effects, including the sexual consequences of their treatment (Davidson *et al*, 2003).

The development of services that improve the patient experience of cancer care and enhance both recovery and quality of life is also endorsed by the Department of Health's latest cancer strategy focused on improving outcomes (Department of Health, 2011, p 50–51). To achieve improvements in the patient experience of survivorship care both policy documents endorse the need to achieve five important service shifts:

- a cultural shift in the approach to care and support for people affected by cancer – to a greater focus on recovery, health and well-being after cancer treatment;
- a shift towards assessment, information provision and personalised care planning;
- a shift towards support for self-management, based on individual needs and with the appropriate clinical assessment, support and treatment;
- a shift from a single model of clinical follow up to tailored support that enables early recognition of and preparation for the consequences of treatment as well as early recognition of signs and symptoms of further disease;
- a shift from an emphasis on measuring clinical activity to a new emphasis on measuring experience and outcomes for cancer survivors through routine use of Patient Reported Outcome Measures (PROMs) in aftercare services.

Achieving such significant cultural and organisational change in oncology is undoubtedly challenging, particularly within current financial constraints. Developing staff and services to deliver new models of aftercare that are characterised by tailored support and personalised care pathways based on an individual's health status, treatment consequences, relationship and life priorities could finally lead to improved sexual rehabilitation for women after cancer.

## Figures and Tables

**Table 1:** Patient demographics in observed consultations

**Patient demographics**	**No. (%) (*n*=69)**
Cervical cancer	20 (29%)
Endometrial cancer	30 (43.5%)
Anal cancer	5 (7.2%)
Rectal cancer	14 (20.3%)
	
*Clinical stage*	
I/II	29 (43.3%)
*Clinical stage*	
III/IV	38 (56.7%)
	
*Treatment type*	
CTRT	31 (44.9%)
EBBRA	32 (46.4%)
EBRT	6 (8.7%)
	
Time post-RT <6 months	31 (44.9%)
Time post-RT 6–11 months	9 (13%)
Time post-RT ⩾12 months	29 (42%)
	
Age of woman ⩽60 years	32 (46.4%)
Age of woman >60 years	37 (53.6%)
	
*Relationship status*	
Partner	48 (69.6%)
No partner	14 (20.3%)
Status not known	7 (10.1%)
	
*Woman accompanied at consultation*	
Yes	30 (43.5%)
No	39 (56.5%)

Abbreviations: CTRT, chemoradiotherapy; EBRT, external beam pelvic radiotherapy; EBBRA, external beam radiotherapy and vaginal brachytherapy.

**Table 2:** Strategies to enhance discussion of treatment-induced female sexual morbidity in oncology practice

**Perceived barrier**	**Practice recommendation**
Clinician embarrassment	Advanced communication skills training Clinical supervision (group) and case discussions Training in psychosexual medicine
	
Lack of knowledge/skills in the assessment of female sexual dysfunction	Development of PROM for treatment-related female sexual morbidity Use of structured patient self-report questionnaires in oncology follow-up to guide consultation agenda Staff training on sexual history taking
	
Lack of knowledge in management of treatment-induced sexual difficulties	Development of clinical guidelines for commonly encountered female sexual difficulties, including sexual aversion/fear, reduced/absent desire, sexual pain, arousal and orgasmic disorders and reduced sexual satisfaction Training in psychosexual medicine
	
Lack of knowledge of specialist services for sexual dysfunction	Development of information resource for patients and clinicians regarding websites, patient information resources and local sexual counselling services Development of agreed clinical management pathways and referral routes within/beyond the cancer centre
	
Inadequate resources/time to address sexual concerns in routine medical follow-up	Development of advanced practice nursing roles for high-risk patient groups (breast, colorectal, gynae-oncology, urology services) Establish nurse-led survivorship programmes/services for range of treatment consequences, including sexual dysfunction Development of psychosexual practice within psycho-oncology services Development of cancer survivorship expertise in primary care roles/services

Abbreviations: PROM, patient reported outcome measures.
